# 3D assessment of a coral reef at Lalo Atoll reveals varying responses of habitat metrics following a catastrophic hurricane

**DOI:** 10.1038/s41598-021-91509-4

**Published:** 2021-06-08

**Authors:** Kailey H. Pascoe, Atsuko Fukunaga, Randall K. Kosaki, John H. R. Burns

**Affiliations:** 1 Marine Science Department, University of Hawai’i at Hilo, Hilo, HI 96720 USA; 2 Joint Institute for Marine and Atmospheric Research, University of Hawai’i aat Manoa, Honolulu, HI 96822 USA; 3 Papahanaumokuakea Marine National Monument, Office of National Marine Sanctuaries, National Ocean Service, National Oceanic and Atmospheric Administration, Honolulu, HI 96818 USA

**Keywords:** Marine biology, Ecology

## Abstract

Extreme disturbances such as hurricanes can cause reductions in coral cover and three-dimensional (3D) structural complexity of coral reefs. We examined changes in structural complexity utilizing 3D reconstruction of a coral-reef site before and after Hurricane Walaka passed through Lalo of the Northwestern Hawaiian Islands. This event resulted in complete destruction of the coral-reef habitat, with dramatic changes in benthic cover from pre-hurricane tabulate coral to post-hurricane rubble. Rugosity and mean slope decreased after the hurricane, while structural complexity, captured by vector ruggedness measure (VRM), showed resolution-specific responses. This metric captured the structural complexity of rubble at a high raster resolution of 1 cm and that of tabulate coral at lower resolutions, resulting in decreases in mean VRM values at 2- and 4-cm resolutions but an increase at 1-cm resolution. Variability in profile and planform curvature was reduced after the hurricane due to a disappearance of extreme curvature values created by the tabulate coral after the hurricane. This study highlights the varying responses of habitat complexity metrics to the complete destruction of a coral reef and provides us with insights into how choices of habitat complexity metrics can affect quantitative assessments of 3D habitat structure.

## Introduction

Extreme natural disturbance events, such as hurricanes and cyclones, are projected to increase in frequency and severity in the future^1^.The waves generated by hurricanes are larger and more powerful than those experienced under normal conditions and are the primary cause of hurricane-related damage to coral reefs^2–5^. For example, Hurricane Allen was one of the strongest recorded Category 5 hurricanes in the Caribbean and produced waves up to 12-m high causing significant damage to shallow-water coral reefs^2^. Loss of live coral cover and habitat structural complexity caused by such disturbance events can lead to detrimental losses in the abundance and diversity of reef organisms, which in turn impact the health and function of coral reef ecosystems^6,7^. Long-term monitoring of coral reefs allows for pre- and post-disturbance comparisons and can be used to assess reductions in the abundance of live coral cover and resulting declines in the three-dimensional (3D) structural complexity of coral reefs^8^.

The vulnerability of corals to storms differs among species, with delicate morphologies typically becoming increasingly vulnerable to dislodgment and mortality as they grow in size^9,10^. Structurally complex branching or tabulate corals are generally more vulnerable to wave damage than corals with more robust and stable growth forms, such as mounding and encrusting corals^11^. In addition, coral communities that exhibit high diversity and richness often show a greater resistance to physical disturbance than monotypic communities^7,12^. This is especially concerning for areas with low species richness and diversity, such as the Hawaiian archipelago. Due to the isolation of the Hawaiian Islands, coral reefs in this area show lower species richness and higher endemism than those found in Indo-Pacific or Caribbean^13^. Thus, Hawai ‘i’s monotypic reef habitats that are comprised primarily of coral colonies with delicate morphologies are of particular interest when assessing the potential impacts of storm disturbances.

Historically, coral reef ecosystems in the Northwestern Hawaiian Islands (NWHI), contained within the Papahānaumokuākea Marine National Monument, were rarely in the track of tropical storms and hurricanes^13^. In October 2018, Hurricane Walaka, which is ranked as the second strongest tropical cyclone in the Central Pacific to date, passed through the NWHI reaching and travelling along a high latitude of 23° North^14,15^. Walaka originated from an area of low pressure south-southeast of Hawai‘i Island and strengthened as it entered the Central Pacific, eventually becoming a Category 5 hurricane on the Saffir-Simpson Hurricane Scale. Hurricane Walaka produced maximum high winds of 205 km/h, and the wave heights associated with the hurricane while it passed through Lalo (French Frigate Shoals) in the NWHI on 3 October 2018 are estimated to have been 11–15 m (per comms. Central Pacific Hurricane Center). The hurricane caused severe damage to coral reefs on the southern end of the shoal and destroyed a unpopulated island of Lalo named East Island^14,15^.

**Figure 1:**
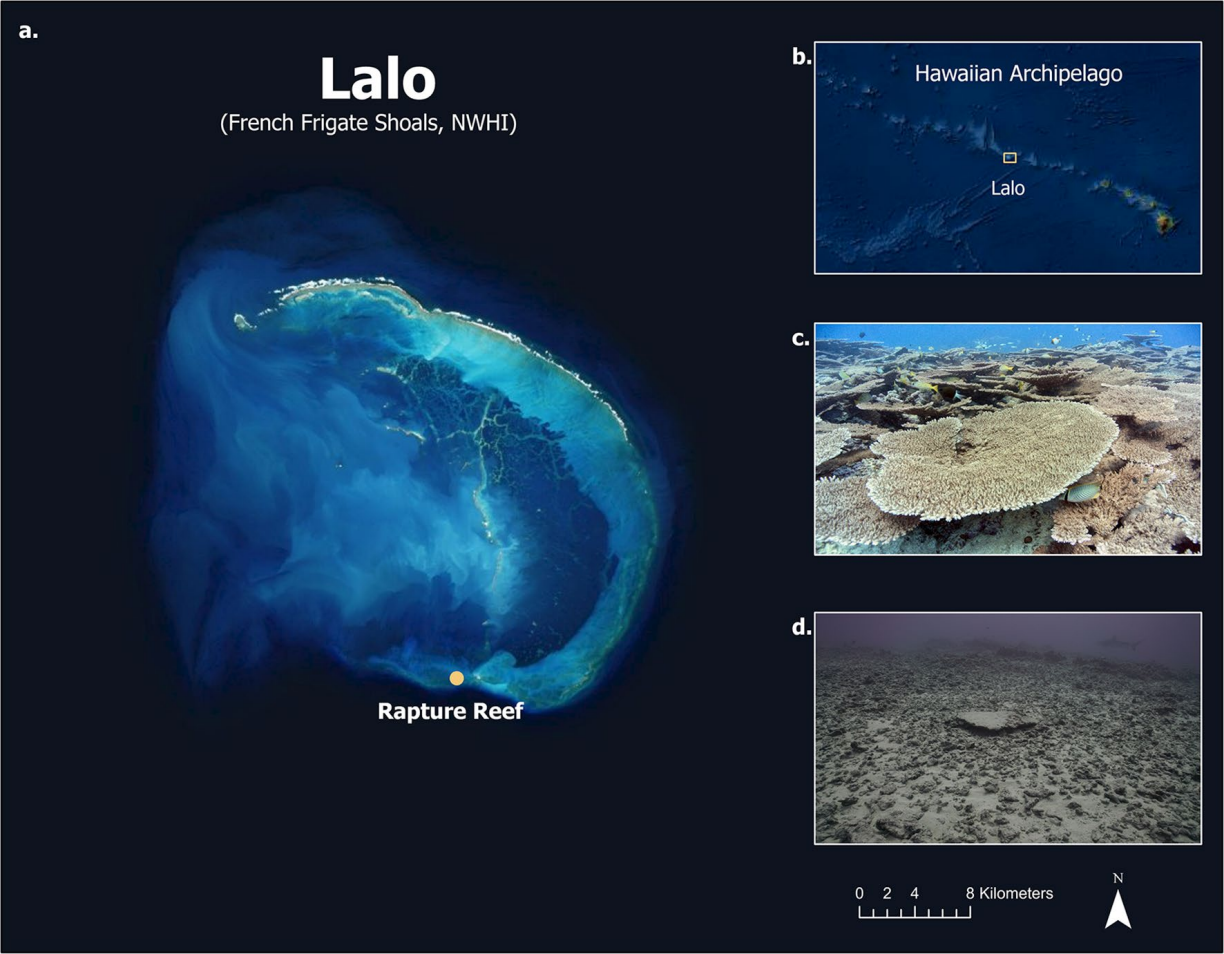
Aerial imagery of (**a**) Rapture Reef, Lalo (French Frigate Shoals) within the Northwestern Hawaiian Islands. Inset map of (**b**) the Hawaiian Archipelago and Lalo’s location. Images showing coral and fish abundance at Rapture Reef (**c**) before (September 2017) and (**d**) after (July 2019) Hurricane Walaka.

We applied photogrammetric techniques to quantify structural changes caused by Hurricane Walaka to an iconic coral-reef study site referred to as Rapture Reef (Fig. 1; 23.63509° N, 166.1857° W) on the southern end of Lalo. Due to their isolation, scientists rarely have the opportunity to thoroughly document impacts of hurricane and storm events on the coral reef ecosystems of the NWHI. Our existing 3D reconstruction data collected from Rapture Reef in 2017, as part of a long-term reef morning project, provided us with a unique opportunity to quantitatively examine changes in benthic cover and 3D habitat structure before and after the hurricane using the high-resolution habitat models. We examined 3D habitat metrics at specific resolutions using analytical methods that have been validated in previous studies to capture habitat architecture created by live corals^16,17^. The main objective of this study was to examine how different habitat metrics responded to the complete destruction of the study site. The photogrammetry and data processing techniques presented here are valuable to any marine ecologists requiring detailed quantitative analyses of changes occurring to 3D habitat complexity and can be easily adapted into existing coral reef monitoring efforts.

## Materials and methods

### 3D photogrammetry surveys

Rapture Reef is located at 20 ~ 30-m depth on the southern end of Lalo, which is the largest atoll in the NWHI with a crescent-shaped reef that stretches approximately 32 km (Fig. 1). Prior to Hurricane Walaka, Rapture Reef was well known for having high cover of tabulate *Acropora* coral that supported an abundant and diverse population of reef fish^18,19^. This site has permanent pins at 26-m depth that are used to facilitate long-term monitoring for several research objectives. Overlapping imagery were collected to monitor a 10 × 5-m^2^ plot at this long-term monitoring site in September 2017 and July 2019 (i.e. before and after the hurricane on 3 October 2018). Images were taken from a planar angle approximately 1 m above the substrata in a boustrophedonic (lawn mower) pattern with 70 ~ 80% overlap between images (see Table 1 for details of camera specifications and settings). Scale bars with coded targets were placed at the corners of the plot for orthorectification of the resulting 3D reconstructions.

**Table 1:** Camera specifications and settings used for photogrammetry surveys.

Camera specifications and settings	
Camera	Canon 5D III (2017), Sony Alpha 7 IIIR (2019)
Camera Lens	24-70 mm
Dome Port	8 in
Focal Length	24 mm
Shutter Speed	1/250 s
ISO	Auto ISO
Aperture	f/10

Structure-from-motion (SfM) photogrammetry methods were used to create 3D reconstructions of the coral-reef plot before and after the hurricane using Agisoft Photoscan/Metashape Professional software (Agisoft LLC., St. Petersburg. Russia) following the methods described in Burns et al.^20^ (Table 2). The software used the coded targets on the scale bars to assign reference points to scale the models to a known local coordinate system. After self-calibrating bundle adjustments were completed using the known reference points to optimize the alignment of images, a dense point cloud and solid triangulated mesh were rendered. An orthophotomosaic and a Digital Elevation Model (DEM) were then generated for each survey year and used for further analysis in CoralNet website (coralnet.ucsd.edu) and statistical software R v.3.5.3 (R core team 2019), respectively^21^.

**Table 2:** Settings used to generate 3D models in Agisoft Photoscan/Metashape Professional software.

Process	Settings
Align photos	High accuracy, generic preselection enabled, 50,000 key point limit, 5,000 tie point limit
Optimize camera alignment	Use all the ones selected by the software
Build dense cloud	Medium quality, mild depth filtering, reuse depth maps disabled
Build mesh	Arbitrary surface type, high face count, interpolation enabled, calculate vertex colors enabled
Build texture	Adaptive orthophoto mapping mode, mosaic blending mode, texture size/count 16,384, enable hole filling

### Quantification of benthic cover

Two-dimensional orthophotomosaics were imported into CoralNet website to compute the percent cover of corals, other invertebrates, algae and abiotic features. For each orthophotomoasic, 1000 random points were generated for manual annotation without any assistance from CoralNet’s auto-annotation function. All live coral were identified down to genus and morphology. Percent cover was obtained for each survey by dividing the number of points for each category by the total annotation points. Annotation points that landed on a transect tape, scale bars and mobile fauna were removed from total number of annotation points.

### Quantification of structural complexity

DEMs were exported at 1-cm raster resolution, which was determined to be the smallest possible cell size while ensuring to be reasonably well within the range of model accuracy (i.e. millimeter-level accuracy) as reported in the results by the ground sampling distance and Root Mean Squared Error (RMSE) values obtained from ground control points (i.e. objects/features of known distances/dimensions) in the models. Both DEMs were processed in R using raster^22^ and rgeos^23^ packages following the procedure of Fukunaga et al.^24^ to obtain the following habitat metrics (Data S1): surface complexity, slope, vector ruggedness measure (VRM) and profile and planform curvature^22–24^. We extracted habitat metrics from 2.5-dimensional DEMs, which captured the reef habitats from a single projected overhead angle, rather than Digital Surface Models (DSMs) due to the computational difficulties associated with quantifying 3D mesh surfaces. Analyzing DEMs is a conventional approach for geospatial analyses of topographic features, and our recent research has found the structural metrics used in this study to be highly correlated when extracted from either a DEM or DSM^17^.

Surface complexity is analogous to reef rugosity measured by the classic chain-and-tape method and is calculated for each DEM as the ratio of the total 3D surface area to 2D planar area^25^. Slope measures an elevational change in degree (i.e. steepness) for each cell of a DEM using 3 × 3 raster cell windows (i.e. calculation of each cell value is based on the elevation value of the cell and those of the surrounding eight cells)^26,27^. Vector ruggedness measure is a computation of surface ruggedness based on the variability in the surface directions of DEM cells, which are determined by a combination of slope and aspect (face direction), and is calculated for each cell using 3 × 3 cell windows^27,28^. Curvature measures the rate of change in slope; profile curvature looks at the direction parallel to slope and planform curvature looks at the direction perpendicular to slope^29^. Similar to slope and VRM, profile and planform curvature are calculated for each cell of a DEM using 3 × 3 cell windows.

All habitat metrics were computed at 1-cm DEM resolution. Additionally, the aggregate function in the raster package was used to calculate VRM at 2- and 4-cm resolutions as VRM at these resolutions have varying capacity to capture the structural complexity of certain coral morphologies, other sessile benthic organisms or topographic features^16,17^. For example, this metric captures the structural complexity of branching corals at a high resolution of 1 cm as the resolution is fine enough to capture coral branches^17^. The structural complexity of mounding corals is, on the other hand, better captured at a low resolution of 4 cm, as this resolution can capture the smooth convex surface of mounding morphology well and is course enough not to be affected by finer structures (e.g. coral branches)^17^. The habitat metrics computed in the present study were, therefore, reef rugosity at 1-cm resolution, slope at 1-cm resolution, VRM at 1-cm, 2-cm and 4-cm resolutions and profile and planform curvature at 1-cm resolution. We aimed to capture the architectural complexity of branching and encrusting corals with VRM at 1- or 2-cm resolution and the complexity of mounding and tabulate corals with VRM at 4-cm resolution^16^. Curvature was included as this metric has the potential to capture the structural complexity of the survey plot created by surface topography such as holes and small ledge-like structure^17^.

The use of DEMs allowed us to utilize the entire DEM cells to calculate habitat metrics for the given area (i.e. the reef plot), thus the entire distribution of each habitat metric could be compared before and after the hurricane. We did not use any statistical tests to compare these values as we obtained the habitat metrics utilizing the entire population (i.e. every single cell of the DEMs) within the reef plot, not random draws of samples from the population. The mean and standard deviation values reported in the present study are the true mean and standard deviation values of the population, not their estimates based on sample mean and standard deviation values, thus any changes in these values before and after the hurricane are real changes that occurred within the reef plot.

## Results

**Figure 2:**
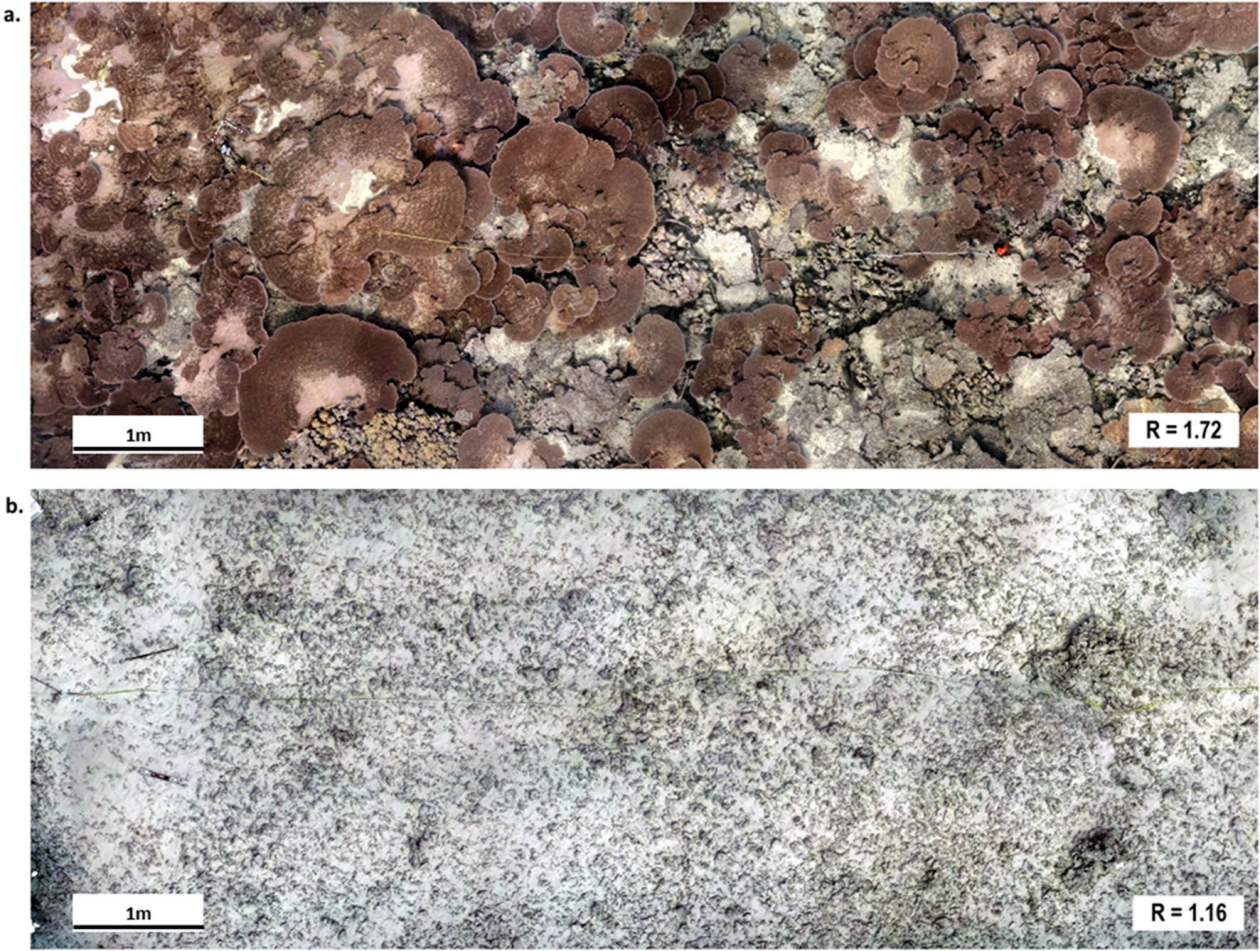
Orthophotomosaic (10 × 5-m) of the coral reef habitat and reef rugosity measurements (R) at Rapture Reef (**a**) before (September 2017) and (**b**) after (July 2019) Hurricane Walaka.

The change in the benthic community of Rapture Reef before and after the hurricane was striking and visually apparent in site photographs (Fig. 1) and orthophotomosaics generated from the 3D reconstruction of the study site (Fig. 2). These images highlight the dominance of live *Acropora* coral on the benthos before the hurricane, while no live corals are evident after the hurricane. Numerically, the survey plot was comprised of 69.7% tabulate *Acropora* (mostly *A. cytherea*), 0.8% encrusting *Porites*, 0.1% encrusting *Montipora*, 1.7% crustose coralline algae, 0.7% sand and 27.0% turf algae on hard substrata before the hurricane. Benthic cover after the hurricane was 67.9% turf algae on rubble and 31.8% sand. There were no other invertebrates detected in the survey year 2017 or 2019.

**Figure 3:**
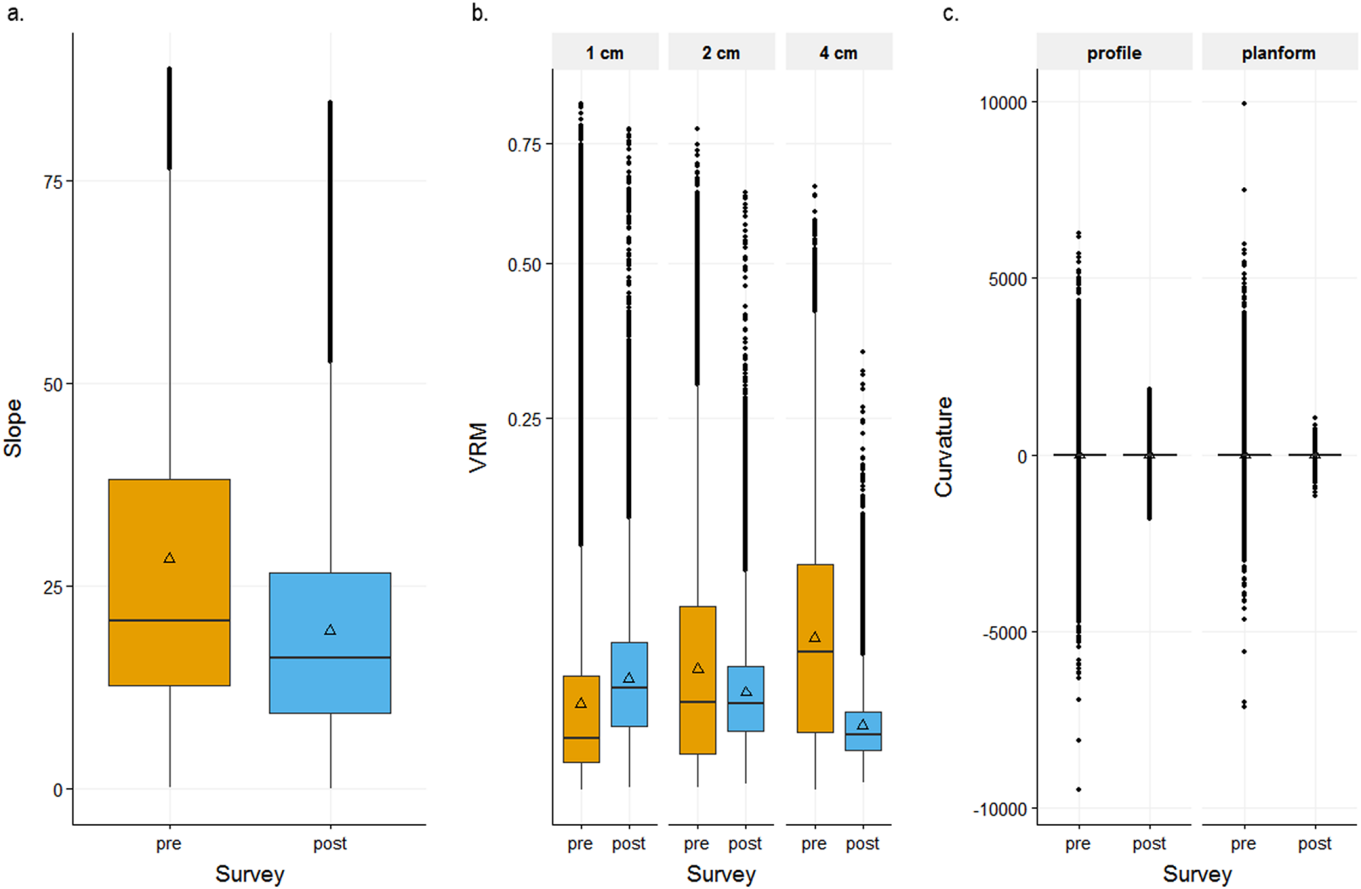
Boxplots of DEM cell values for each habitat metric before (pre) and after (post) Hurricane Walaka, showing the ranges of (**a**) slope, (**b**) terrain ruggedness measure (VRM) at 1-, 2- and 4-cm resolutions with the y axis in square-root scale and (**c**) planform and profile curvature. Mean values are displayed with a triangle symbol within each boxplot.

**Figure 4:**
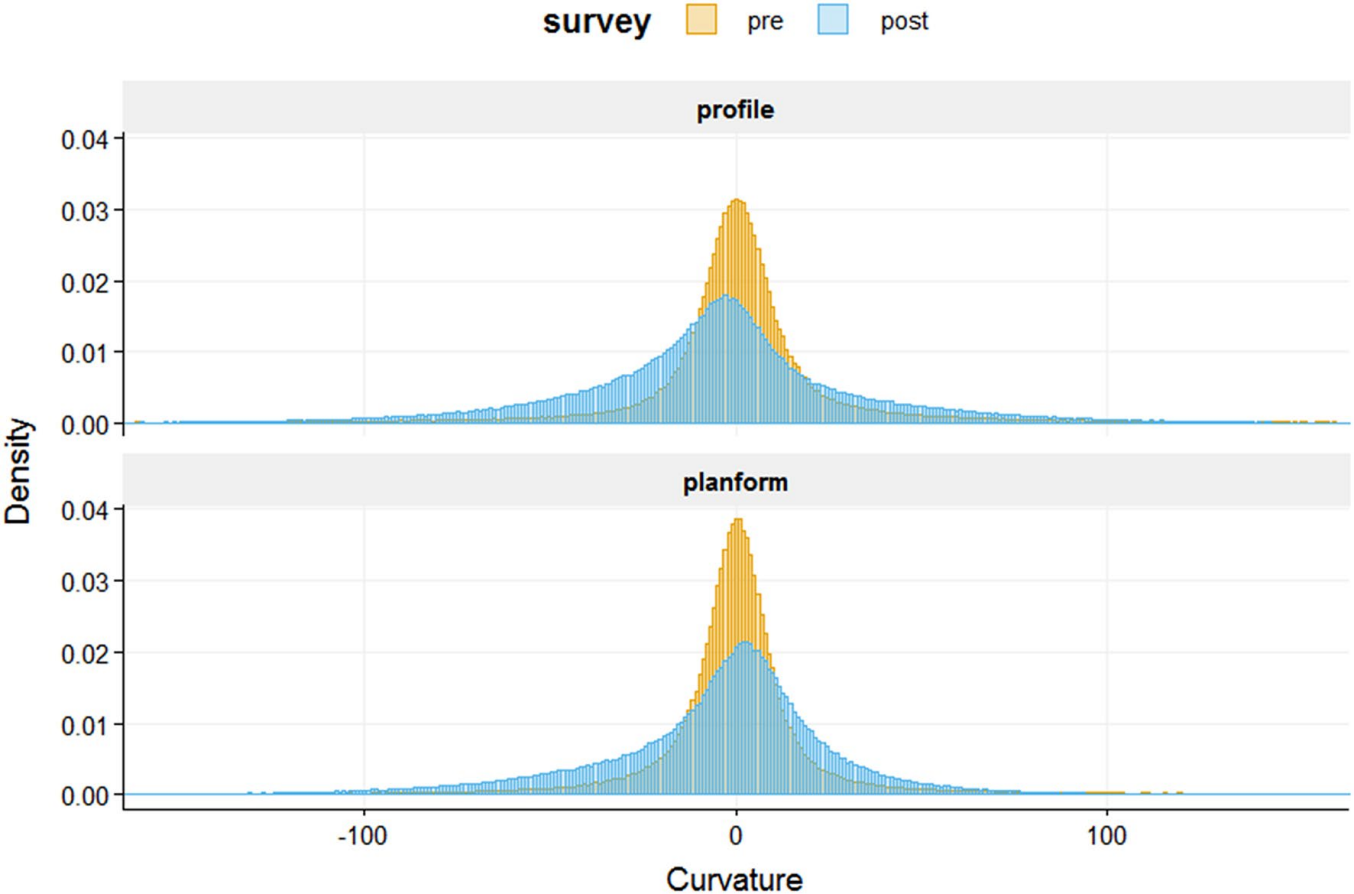
Density plot showing distribution of planform and profile curvature values between − 100 and 100 before (pre) and after (post) Hurricane Walaka. Note that the density was calculated for each curvature type using the entire data, not just those between − 100 and 100.

Ground sampling distance (GSD) values (resolution/pixel) for the 3D reconstructions generated for survey years 2017 and 2019 were 0.00128 m/pix and 0.00039 m/pix, with errors of 0.003 pixels and 0.073 pixels, respectively. Root mean standard error (RMSE) values for ground control points for the 2017 and 2019 survey years were 0.00143 m and 0.00136 m, respectively. Reef rugosity obtained at 1-cm DEM resolution decreased from 1.72 to 1.16 following Hurricane Walaka (Fig. 2). The mean value of slope also decreased from 28.13 to 19.44, as well as the overall range and the standard deviation (SD) from 21.50 to 13.38 (Fig. 3a). Similarly, mean VRM values at 2- and 4-cm resolutions decreased from 0.045 (SD = 0.069) to 0.023 (SD = 0.028) and from 0.062 (SD = 0.076) to 0.010 (SD = 0.015), respectively (Fig. 3b). Conversely, mean VRM at 1-cm resolution increased from 0.027 (SD = 0.055) to 0.030 (SD = 0.034) (Fig. 3b). For curvature, mean planform curvature and mean profile curvature decreased from 1.52 to − 5.75 and from 1.58 to − 5.75, respectively, but more notably, the overall ranges of both profile and planform curvature dramatically decreased (Fig. 3c). The distribution of curvature values before the hurricane exhibited higher standard deviation, thus variance (SD = 270.62 and 59.31 before and after the hurricane, respectively, for profile curvature and 127.13 and 38.88 for planform curvature), as well as a higher density of values close to 0 (i.e. flat; Fig. 4) in comparison to values extracted after the hurricane.

## Discussion

This study used SfM photogrammetric techniques to quantify benthic community composition and 3D habitat structural complexity of Rapture Reef before and after Hurricane Walaka. The destruction of live coral at Rapture Reef after the hurricane, particularly the complete loss of the tabulate coral *Acropora spp.*, was apparent from the site photos (Fig. 1) and orthophotomosaics (Fig. 2). As the change in the benthic community composition was so visually obvious, the use of orthophotomosaics to enumerate benthic cover might be deemed unnecessary in this case. Nevertheless, it offered additional quantitative information, particularly about organisms that were not dominant and visually-obvious components of the community (i.e. encrusting corals, crustose coralline algae and turf algae), and the procedure is valuable for other studies that have to deal with subtle changes in benthic community compositions caused by disturbance events.

The loss of *Acropora* coral at Rapture Reef was comparable to the severe damage to shallow coral reefs caused by Hurricane Allen, a Category 5 hurricane, in Jamaica in 1980. Studies conducted after the impact of Hurricane Allen found that 99% of all branching *Acropora* species were damaged on coral reefs at 6-m depth in comparison with 9% mounding and 23% encrusting and plating morphologies^2^. In the present study, while the surf height during Hurricane Walaka could only be estimated without a reliable means to measure, the surf and surge were clearly large enough to obliterate the live coral at a depth of 26 m. Structurally complex and fragile branching or tabulate corals are physically susceptible to breakage from wave actions, and tabulate corals are also easily subjected to full mortality by dislodgement due to their large, top-heavy morphology^11^. The dominance of tabulate *Acropora* prior to the hurricane likely made Rapture Reef particularly susceptible to the impact of the intense wave energy created by the hurricane.

The shift in the benthic community composition at Rapture Reef resulted in changes in the structural complexity of the habitat. An overall decrease in reef rugosity at 1-cm resolution was detected after the hurricane. Decreases in mean slope at 1-cm resolution, mean VRM at 2- and 4-cm resolutions and mean profile and planform curvature at 1-cm resolution were also detected. Conversely, mean VRM at 1-cm resolution increased after the hurricane (Fig. 3). These different metrics possess unique properties in capturing the structural complexity of a coral-reef habitat^17^. Collectively examining metrics of 3D structural complexity provides us with important insights into the impact of Hurricane Walaka on the habitat architecture of Rapture Reef. The decrease in reef rugosity represents an overall decrease in 3D surface area after the hurricane, while the decrease in mean slope is likely due to loss of vertical relief resulting from live corals (mostly tabulate *Acropora*) turning into rubble as slope is the measure of steepness^26,27^ (Table 3).

**Table 3:** Summary table showing properties of different habitat metrics that are recommended for characterization of coral-reef habitats.

3D habitat metric	Resolution	Statistic	Suited to capture	Supporting references	Original references
Surface complexity	1 cm	N/A	Overall changes in 3D surface area relative to 2D planar area	^22^, present study	^23^
Slope	1 cm	Mean	Overall changes in vertical relief (including loss of tabulate and mounding corals)	^22,29^, present study	^24,25^
Vector ruggedness measurement (VRM)	1 or 2 cm	Mean	Structural complexity of branching, encrusting corals, and rubble	^28,29^, present study	^25,26^
	4 cm	Mean	Structural complexity of mounding and tabulate corals	^28,29^, present study	
Profile and planform curvature	1 cm	Range/Variance	Holes and ledges, including the "drops" created by tabulate corals	^29^, present study	^27^
Fractal dimension	1–8 or 16 cm for corals, but range is case dependent	N/A	Overall structural complexity of corals and crustose coralline algae	^22,28,29^	^34,35^

Listed are the recommended habitat metrics and resolutions, statistic (n/a means that there is only a single value produced per DEM), suitable benthic features to capture, references that support this recommendation, and original references of each metric.

VRM quantifies the variability in the slope and aspect of each DEM cell and ranges from 0 (no variation) to 1 (highest variation), with most natural environments exhibiting VRM values of less than 0.40^28^. The resolution-specific response of the VRM metric (Fig. 3b) was consistent with our previous studies where we found the structural complexity of branching corals and rubble being captured at relatively high resolutions, encrusting corals and crustose coralline algae at intermediate resolutions and tabulate and mounding corals at relatively low resolutions^16,17^ (Table 3). The increase in mean VRM at 1-cm resolution after the hurricane in the present study can be explained by high variability in aspects and slopes created by individual pieces of rubble being captured at this resolution. The decrease in the mean VRM at 4-cm resolution was due to the complete loss of *Acropora*, as this lower resolution more effectively captured variability in slope and aspect associated with the overhang structure created by the tabulate coral morphology (Table 3).

Curvature metrics do not monotonically respond to changes in structural complexity, as a flat surface has a curvature value of 0 and the value increases towards either the positive or negative direction depending on the concavity/convexity of the surface^29^. In coral-reef habitats, the juxtaposition of concavity and convexity makes interpretation of mean curvature values difficult. The overall distribution of curvature values obtained from the DEM offers more meaningful information for interpreting the complexity of coral-reef habitats^17^ (Table 3). In the present study, the variance in curvature values at 1-cm resolution dramatically decreased following Hurricane Walaka, with extreme positive and negative values being observed in the pre-hurricane DEM disappearing in the post-hurricane DEM (Fig. 3c). Such extreme curvature values derive from sudden changes in slope and are likely generated by the tabulate *Acropora* coral in the present study^17^. As our DEMs were rendered from a planar perspective, an abrupt drop in elevation and sudden change in slope occurs at the edge of the table-top structure of the *Acropora* coral (Table 3). Tabulate corals can also create the illusion of leveled (i.e. flat) surface, as 1-cm resolution is too coarse to capture the fine-scale structures of small interlacing branches and tube-like corallites. This can explain the higher proportion of DEM cells having curvature values closer to 0 before the hurricane than after the hurricane (Fig. 4).

The present study quantified structural damage caused by Hurricane Walaka at a single site of Lalo (i.e. Rapture Reef) and was not designed to assess the overall impacts of the hurricane. Rather, with the presence of pre-hurricane 3D reconstruction from a long-term monitoring project, it utilized the unique opportunity to determine how the complete destruction of the living coral community and entire loss of aggregate carbonate reef structure affected metrics of 3D structural complexity. The entire loss of aggregate carbonate habitat presents a novel scenario as other studies examining changes in 3D habitat structure following large-scale coral mortality studied habitats that retained the carbonate reef structure from the coral skeletons^8,30,31^. The use of the DEMs rendered from the planar (overhead) angle in the present study meant that the habitat created under the tabulate *Acropora* at the reef site (mostly *A. cytherea*) would not be effectively captured in the DEMs. Nevertheless, the presence of *Acropora* in the pre-hurricane plot and its loss in the post-hurricane plot were sufficiently captured by the changes in mean slope, mean VRM at 4-cm resolution and the range in curvature values. The varying (i.e. resolution-specific) responses of VRM also highlights the importance of understanding the unique properties of different habitat metrics; if VRM at 1-cm resolution was the only metric to be used to quantify the changes in the structural complexity before and after the hurricane, we would have reached, counterintuitively, a conclusion of an increase in the structural complexity of the reef site. This study illustrates the value of examining metrics of 3D habitat complexity across a range of scales and resolutions to accurately measure how changes in benthic composition will impact the architectural complexity of coral reef habitats, and in turn, affect reef biodiversity. Table 3, which synthesizes and summarizes findings from our previous and current studies, should provide some guidance to identify appropriate habitat metrics and resolutions to quantify specific habitat features.

The importance of reef rugosity or surface complexity to the health and function of coral-reef ecosystems has been known for decades, with many studies documenting positive associations between the structural complexity of habitats and reef fish assemblages^6,25,32,33^. *Acropora cytherea* creates structurally complex habitats due to the distinct tabulate morphology and associated overhangs where reef fishes are often found in high abundance^19,20^. Despite the high abundance and diversity of reef fish at Rapture Reef prior to the hurricane^18,19^, no reef fish was observed at the site during the 2019 survey with the exception of a single shark (K.H.P. personal observation), highlighting the potentially devastating impact of the loss of structural complexity and microhabitat created by tabulate *Acropora* on the reef ecosystem. The post-hurricane data in the present study can now serve as baseline data to document the potential recovery of iconic Rapture Reef in coming years, being contingent upon successful coral recruitment onto the unstable substratum consisting of unconsolidated rubble. Proper understanding of unique properties of different 3D habitat metrics, with varying capacity to capture the structural complexity of different morphologies and topographic features, is vital when quantifying the structural changes in coral-reef habitats over time. As extreme weather events are expected to increase in frequency and severity due to climate change, such understanding is essential to continuous long-term monitoring and assessment of ecologically, economically, and culturally important coral reef ecosystems.

## Supplementary Information

Supplementary Information.
