# Supplementary material for: 3D assessment of a coral reef at Lalo Atoll reveals varying responses of habitat metrics following a catastrophic hurricane
Source: Sci Rep. 2021 Jun 8;11:12050. doi: 10.1038/s41598-021-91509-4 (PMC8187721; doi:10.1038/s41598-021-91509-4)
Supplement: Supplementary file 1 — Supplementary Information. [file 41598_2021_91509_MOESM1_ESM.zip › Data_S1/Data_S1_Cover_Page.docx]

3D assessment of a coral reef at Lalo Atoll reveals varying responses of habitat metrics following a catastrophic hurricane

**Authors:** Kailey H. Pascoe^1*^, Atsuko Fukunaga^2,3^, Randall K. Kosaki^3^, John H. R. Burns^1^

**Affiliations:**

1. Marine Science Department, University of Hawai’i at Hilo, Hilo, HI 96720, USA; johnhr@hawaii.edu
2. Joint Institute for Marine and Atmospheric Research, University of Hawai’i at Manoa,

Honolulu, HI 96822, USA; [atsuko.fukunaga@noaa.gov](mailto:atsuko.fukunaga@noaa.gov)

1. Papahanaumokuakea Marine National Monument, Office of National Marine Sanctuaries, National Ocean Service, National Oceanic and Atmospheric Administration, Honolulu, HI 96818, USA; randall.kosaki@noaa.gov

* Correspondence: kpascoe@hawaii.edu; Tel.: +1-808-351-4483

Supplementary Data Contents:

**Data_S1.R**: This is the R script used to extract 3D habitat metrics from the before and after Digital Elevation Models (DEMs) tiff files.

**Pre_walaka_dem_1cm.tiff**: DEM file taken before hurricane Walaka passed through Lalo Atoll. This DEM will be analyzed through the Data_S1 r script. This file is not visible through photo viewers because of the floating point information of the digital elevation model. This can be viewed in most mapping or 3D programs (i.e., ArcGIS or Agisoft Software)

**Post_walaka_dem_1cm.tiff**: DEM file taken after the hurricane Walaka passed through Lalo Atoll. This DEM will be analyzed through the Data_S1 r script. This file is not visible through photo viewers because of the floating point information of the digital elevation model. This can be viewed in most mapping or 3D programs (i.e., ArcGIS or Aigsoft Software)

**Pre_walaka_dem_1cm.jpeg:** Visible jpeg file of the DEM taken before hurricane Walaka passed through Lalo Atoll. This file cannot be analyzed in the R scripts because it does not support the floating point information of the DEM.

**Post_walaka_dem_1cm.jpeg:** Visible jpeg file of the DEM taken after hurricane Walaka passed through Lalo Atoll. This file cannot be analyzed in the R scripts because it does not support the floating point information of the DEM.
