# Supplementary figures and images for: 3D assessment of a coral reef at Lalo Atoll reveals varying responses of habitat metrics following a catastrophic hurricane
Source: Sci Rep. 2021 Jun 8;11:12050. doi: 10.1038/s41598-021-91509-4 (PMC8187721; doi:10.1038/s41598-021-91509-4)

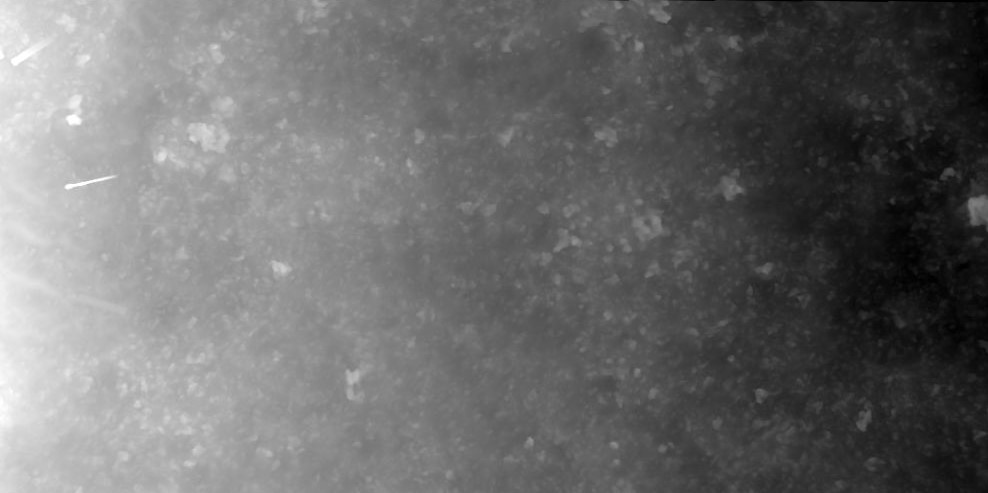

Supplement: Supplementary file 1 — Supplementary Information. [file 41598_2021_91509_MOESM1_ESM.zip › Data_S1/post_walaka_dem_1cm.jpg]

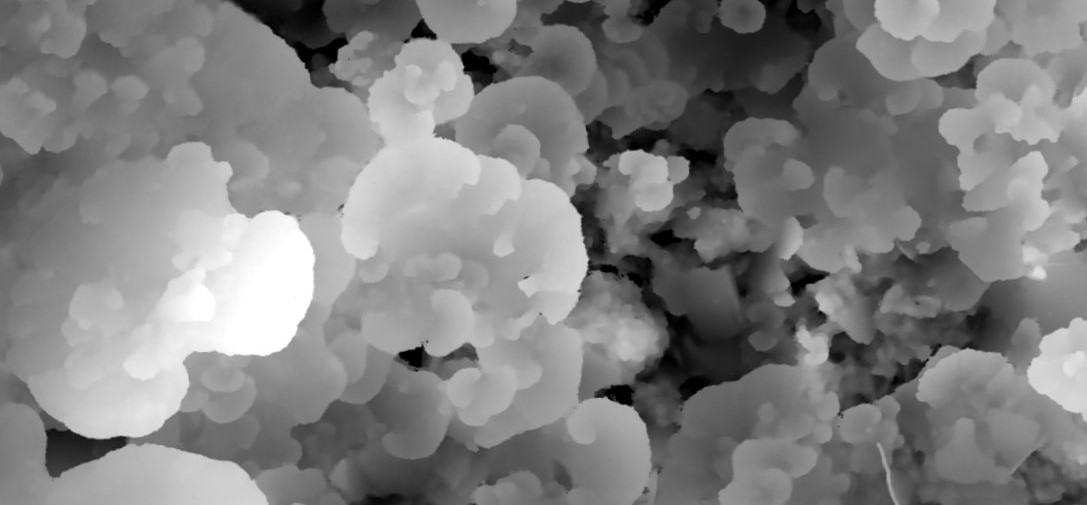

Supplement: Supplementary file 1 — Supplementary Information. [file 41598_2021_91509_MOESM1_ESM.zip › Data_S1/pre_walaka_dem_1cm.jpg]
